# Activity of *Lactobacillus gasseri* strain LLG1 and its exopolysaccharides in an inflammatory cell model and on *Candida albicans* proliferation

**DOI:** 10.1080/21505594.2026.2707757

**Published:** 2026-08-28

**Authors:** Rogatien Charlet, Karine Lecointe, Oksana Plu, Camille Cordier, Clovis Bortolus, Pierre Popczyk, Faustine Dubar, Sébastien Grec, Boualem Sendid

**Affiliations:** a CNRS, UMR 8576 - UGSF - Unité de Glycobiologie Structurale et Fonctionnelle, University Lille, Lille, France; b INSERM U1285, University Lille, Lille, France; c CHU Lille, Laboratoire de Parasitologie-Mycologie, Lille, France

**Keywords:** *Lactobacillus gasseri*, exopolysaccharides, *Candida albicans*, biofilm formation, inflammation, immune modulation

## Abstract

Exopolysaccharides (EPS) are carbohydrate polymers produced by various bacteria, including species of the genus *Lactobacillus*. Until now, the link between the functional properties of these bacteria and their EPS remains poorly understood. Lactobacilli are used in a wide range of applications due to their protective effects against inflammation and their role in maintaining intestinal homeostasis. However, the functional properties of their EPS remain weakly characterized. This study investigated the EPS produced by *Lactobacillus gasseri* (*Lg*-EPS), a human-derived probiotic, focusing on their immunomodulatory activity and inhibitory effects on the growth and virulence of *Candida albicans*, an opportunistic fungal pathogen associated with dysbiosis and immune dysfunction. The results show that *Lg*-EPS are composed of 59.1% glucose, 26.0% galactose, 14.6% rhamnose, and 0.3% N-acetyl-glucosamine. Functionally, *Lg*-EPS reduced the expression of pro-inflammatory cytokines (IL-6, IL-8, CCL-2) and IL-6 protein levels, while increasing IL-10 expression and secretion. These effects were associated with downregulation of TLRs, MyD88 and NF-κB, alongside upregulation of the aryl hydrocarbon receptor (AhR), an intracellular transcription factor involved in immune modulation. Additionally, *Lg*-EPS increase induction of AhR activity in HT29-Lucia™ AhR cells. Furthermore, *Lg*-EPS inhibited *C. albicans* biofilm formation and fungal growth. These findings suggest that *Lg*-EPS contribute to immune regulation and antifungal defense *via* AhR activation. This receptor, known for sensing microbial metabolites, mediates anti-inflammatory effects by suppressing NF-κB signaling, enhancing IL-10 production, and reinforcing epithelial barrier function. This is the first report to demonstrate the AhR-dependent anti-inflammatory effect of bacterial EPS.

## Introduction

Colonization of the digestive tract with commensal flora, after birth, establishes a permanent and important dialogue with the host which is essential for maturation of the immune system [1]. Lactic acid bacteria, including *Lactobacillus* and *Bifidobacterium* spp., are among the earliest microbial colonizers of the infant gastrointestinal (GI) tract. These microorganisms exert protective effects against pathogenic bacteria [2,3]. This activity is attributed to the production of selective metabolic compounds, including saccharolytic and acidogenic agents, as well as the synthesis of bacteriocins, lytic enzymes, and organic acids with antimicrobial properties [3].

*Lactobacillus gasseri* is a probiotic species and is one of the predominant commensal microorganisms in the human GI tract [4,5] and the female genital tract [6]. *L. gasseri* has been associated with a range of antimicrobial [7,8], anti-carcinogenic [9] and anti-inflammatory activities [10,11]. A recent investigation using a murine model of ulcerative colitis demonstrated that daily intragastric administration of *L. gasseri* significantly mitigated intestinal inflammation [12]. This anti-inflammatory effect was demonstrated by reductions in histological scores, myeloperoxidase activity, and pro-inflammatory cytokine levels.

Although the pathogenesis of chronic inflammatory bowel diseases (IBDs) is unknown, these diseases appear to be multifactorial, with genetic and environmental factors capable of altering the intestinal equilibrium and inducing a deregulation of the immune response to components of the intestinal microbiota [13–15]. These diseases may also be the origin of colorectal cancer, linked to inflammatory factors such as reactive oxygen species (ROS), pro-inflammatory cytokines, and chemokines contributing to tumor cell growth and spread [16,17]. Patient management primarily involves controlling the active phases of the disease through medical treatment, with the objective of prolonging periods of remission and promoting mucosal healing. However, these treatments may prove inadequate over time due to immunogenicity or the emergence of long-term adverse effects [15,18,19]. Consequently, alternative preventive strategies have been explored, focusing on the administration of probiotic bacteria or their metabolites in order to mitigate the pro-inflammatory responses characteristic of IBDs [5,19–21].

In addition, the gut microbiota also includes fungal flora, which is poorly represented, comprising less than 1% of microorganisms. However, this flora plays a crucial role in maintaining intestinal homeostasis. Among its constituents is *Candida albicans*, a saprophytic commensal yeast of the human digestive tract, which is capable of excessively colonizing the digestive mucosa under certain pathophysiological conditions. For example, alterations in the intestinal bacterial flora, the digestive mucosa, or an immune deficiency can promote the translocation of these yeasts across the digestive epithelial barrier [22,23]. This disruption of the gut microbiota, and more specifically of *Candida* spp., appears to play a decisive role in maintaining the intestinal inflammation associated with IBDs. In this context, our team explored the fungal pathway as a triggering factor or a contributing factor in maintaining intestinal inflammation during IBDs. This work follows the description in the early 1990s of anti-yeast antibodies called ASCA (anti-*Saccharomyces cerevisiae* Antibody) found in more than 60% of patients with Crohn’s disease (CD). Subsequently, these antibodies were correlated with small bowel involvement and a young age at diagnosis [24–26]. Other studies have shown that the magnitude of the ASCA serological responses and antimicrobial antibodies are correlated with disease severity [27,28]. Finally, experimental studies suggest a link between *C. albicans* and the inflammation observed in CD [29,30]. However, it remains difficult to determine whether this is a cause or a consequence of the uncontrolled inflammation that characterizes CD.

During the last decade, our team has investigated the molecular mechanisms governing inflammatory processes, with an emphasis on the contribution of specific members of the gut microbiota to the reduction or exacerbation of inflammation. Bacteria from the murine digestive tract, including *Lactobacillus* spp., have been shown to possess antifungal properties *via* hydrolytic enzymes directed against fungal cell wall components, leading to the inhibition of fungal growth [31]. Significant variations in the content of fatty acids released from the interaction between these bacteria and intestinal epithelial cells have also been demonstrated. More recent studies have investigated the direct antifungal properties of these fatty acids against *Candida* spp., in terms of viability, adherence, fungal growth, and biofilm formation [32]. However, the mechanisms that direct the *Candida-*bacteria interactions and the bacterial metabolites involved have not been widely explored, particularly polysaccharide metabolites and exopolysaccharides (EPS).

This study focused on the extraction and characterization of EPS from *L. gasseri* strain LLG1 (*Lg*-EPS) and their biological properties. *Lg*-EPS play an important role in the probiotic activity of this species, by participating in intestinal homeostasis and host immunity. However, the recognition and mechanisms involved in the action of these metabolites, particularly in the immune response, remain to be clarified. These molecular determinants also play a role in inhibiting the growth of bacterial pathogens such as *Escherichia coli*, while favoring the growth of symbiotic bacteria and the development of bacterial biofilms, with beneficial activity on the digestive immune system [8]. Nevertheless, studies on the inhibitory activity of *Lg*-EPS against the digestive mycobiota, particularly *Candida albicans*, remain scarce.

## Materials and methods

### Bacterial cultures

The bacterial species *L. gasseri* (LLG1, strain collection of Lille teaching Hospital) was used. This strain was initially isolated from the vaginal tract of a pregnant woman and stored at −80°C until further use. In order to identify the bacterial species, colonies were mixed with 1.5 μL matrix solution (α-cyano 4-hydroxycinnamic acid; 8,201,344; Bruker Daltonics) dissolved in 50% acetonitrile, 47.5% water, and 2.5% trifluoroacetic acid. The extract was placed on a target spot on a metallic plate and analyzed by MALDI-TOF MS (Microflex-Bruker Daltonics). Bacterial culture was performed in De Man, Rogosa and Sharpe liquid medium (MRS; CM0359; OXOID) on a rotary shaker for 48 h at 37°C under anaerobic conditions. Culture was carried out in jars containing 2.5 L of anaerobic atmosphere generator (AnaeroGen^TM^; AN0025A; Sigma-Aldrich). The bacterial suspensions obtained were then centrifuged at 1200 rpm for 5 min and washed in phosphate-buffered saline (PBS (10X); 14,200–067; Gibco).

### Extraction of Lg-EPS

EPS were extracted using a protocol adapted from previously described methods [33,34], including NaOH treatment, ethanol precipitation, and dialysis to remove low molecular weight contaminants. Briefly, the bacterial culture was placed in a 100 mL Erlenmeyer flask in MRS medium for 48 h with shaking at 37°C under anaerobic conditions. The culture was washed twice with PBS, centrifuged at 3200 rpm for 10 min and the pellet was recovered and stored at −20°C. 10 mL NaOH was added to the pellet (2 M; S/4880/60; Fisher Chemical) for 1 night with shaking and the mixture was then centrifuged at 5000 g for 30 min. The supernatant was recovered, 20 mL ethanol was added (2:1) and the mixture was left overnight at −20°C. Finally, the extract was centrifuged at 5000 g for 30 min, 2 mL of Milli-Q water was added and dialysis (Dialysis tubing SnakeSkin™; 88,243 10kDa; Thermo Scientific) was performed at 4°C for 48 h. The dialyzate was recovered and the specimen was freeze-dried.

### Evaluation of EPS purity by SDS-PAGE, Bradford assay, spectrophotometer, and Western blot

#### SDS-PAGE and Bradford assay

The EPS were analyzed by 10% SDS-PAGE to verify the absence of proteins in the batches. Before loading the samples into the gel wells, Laemmli buffer was added and thermal denaturation was carried out at 95°C for 5 min. A total of 20 µg *Lg*-EPS was loaded into the wells of the SDS-PAGE gel. The positive control consisting of 20 µg of bovine serum albumin (BSA; A9647-100 G; Sigma) was also dispensed in one well. In parallel, 5 µL molecular weight marker was dispensed into another well (PageRuler Plus Prestained Protein Ladder; 26,619; Thermo Scientific). Electrophoretic separation was carried out in SDS-containing buffer (TG-SDS 10X Tris-glycine-SDS buffer; EU0510-B; Euromedex) until the proteins were completely separated. The gel was stained with PageBlue (PageBlue Protein Staining Solution 1 L; 24,620; Thermo Scientific), which was added for 1 h followed by three washes in Milli-Q water with shaking, and the gel was visualized using the iBright imaging system (iBright 1500; Invitrogen). In parallel, total protein content was quantified using a BCA assay (Pierce^TM^ BCA Protein Assay Kit; 23,227; Thermo Scientific) for quality control purposes. The corresponding data are available online [35].

### Spectrophotometer (nucleic acid contamination)

Nucleic acid contamination was evaluated using a spectrophotometer instrument (DeNovix DS-11) by measuring absorbance at 260 and 280 nm.

### Western blot lipoteichoic acid (LTA)

The presence of LTA in *L. gasseri* and in *Lg*-EPS was analyzed by Western blot according to the protocol described by Millership and Gründling [36]. Briefly, *L. gasseri* or *Lg*-EPS were isolated as described in the sections “bacterial cultures” and “extraction of *Lg*-EPS” of the Materials and Methods. For *Escherichia coli* (EC11303; Sigma-Aldrich; negative control), bacterial culture was performed in MCK (MacConKey; 1,053,960,500; Millipore) on a rotary shaker for 24 h at 37°C. All the bacterial suspensions obtained were centrifuged at 1200 rpm for 5 min, washed in PBS and quantified at 600 nm (10^9^ cells/OD unit). Cells were lysed using 0.1 mm glass beads with a bacteria-specific program (FastPrep-24TM 5 G; MP Biomedicals). Lysates were clarified by centrifugation (200 g for 1 minute to remove the beads and 17,000 g for 10 minutes to precipitate the bacterial debris) and suspended in SDS-PAGE sample buffer normalized to OD600 (absorbance = 0.3 OD unit), before being heated to 100°C for 20 min, and stored at − 20°C or used immediately. In parallel, samples of *Lg*-EPS or LTA control (LTA from staphylococcus; L2515-5 MG; Sigma; positive control) were prepared at a concentration of 1 mg/ml. Briefly, the *Lg*-EPS or LTA were dissolved in milliQ water and vortexed for 60 s. The samples were then incubated at 37°C for 10 minutes before being stored at −20°C or used immediately. A standard 10% SDS-PAGE gel with 10 wells is prepared (as described in the section “SDS-PAGE” of the Materials and Methods). Before loading the samples into the wells, Laemmli buffer was added and thermal denaturation is carried out at 95°C for 5 minutes. The samples obtained (20 µL final volume) were loaded into the wells of the SDS-PAGE gel and 5 µL of pre-stained protein marker was loaded into one well (PageRuler Plus Prestained Protein Ladder; 26,619; Thermo Scientific). The proteins were transferred to a PVDF membrane (PVDF Transfer Membrane; 88,585; Thermo Scientific), which had been pre-activated with methanol (Methanol; 20,846.292; VWR), using electrotransfer (150 V, 300 mA for 1 hour). The membrane was incubated in the blocking solution (Tris-buffered saline with Tween 20; TBST) containing 5% BSA, for 1 h with shaking at room temperature (RT). Incubation with the primary antibody directed against LTA was carried out overnight at 4°C (1:1000; Gram Positive Bacteria LTA monoclonal Antibody (G43J); MA1-7402; Invitrogen). The PVDF membrane was washed three times for 10 min with TBST. A horseradish peroxidase (HRP)-conjugated secondary antibody was then applied (1:5000; Goat anti-mouse IgG (H+L) secondary antibody HRP; 62–6520; Invitrogen) for 1 h at RT. The PVDF membrane was washed three times for 10 min with TBST and detection was then performed by chemiluminescence (SuperSignal West Pico Chemiluminescent Substrate; 34,080; Thermo Scientific) and the signal was visualized using the iBright imaging system (iBright 1500; Invitrogen).

### Monosaccharide composition of Lg-EPS by trimethylsilylation (TMS) and GC-FID

Aliquots (100 μg) of the samples, supplemented with 10 μg meso-inositol as an internal standard, were lyophilized in a glass tube. The samples were then subjected to methanolysis in 500 μL of anhydrous 0.5 M MeOH/HCl (Methanol; 20,846.292; VWR – Hydrochloric acid; Acros Organics) and incubated at 80°C for 24 h. Following methanolysis, the reaction mixtures were neutralized by the gradual addition of silver carbonate (Ag_2_CO_3_; 179,647-5 G; Sigma) until the pH reached 6–7. N-reacetylation was performed by adding 20 μL pure acetic anhydride, followed by overnight incubation at room temperature (RT) in the dark. Tubes were then centrifuged at 2000 g for 5 min and the supernatants were transferred to fresh glass tubes and dried under a stream of nitrogen. Once dried, 25 μL pyridine and 50 μL BSTFA (N, O-Bis(trimethylsilyl)trifluoroacetamide) were added and the mixtures were incubated at RT for 2 h. The derivatized samples were then injected into GC-MS (AGILENT Technologies) and compounds were detected at 70 eV in full scan mode from 45 to 500 Da. Separation was performed on a capillary column (SolGel-1 ms, 30 m × 0.25 mm × 0.25 μm; Trajan Scientific and Medica) using a temperature gradient from 120°C to 240°C at a rate of 2°C/min, followed by a 10 min isothermal hold at 240°C.

### Cell cultures

Cell cultures were prepared using Caco-2 cells, a human epithelial cell line of intestinal origin. Culture was carried out in Dulbecco’s modified eagle medium (DMEM) containing 20% fetal bovine serum (FBS; S1810-500; biowest) and 100 U/ml-100 µg/mL of penicillin-streptomycin (100 mL; 15,070–063; Gibco). HT29-Lucia^TM^ AhR cells were also used (AhR reporter cells; ht2l-ahr; InvivoGen), in DMEM containing 10% FBS, 100 U/ml-100 µg/mL of penicillin-streptomycin, 100 µg/mL of Normocin^TM^ (ant-nr-05; InvivoGen) and 100 µg/ml of Zeocin^TM^ (selective antibiotics; ant-zn-1; InvivoGen). THP-1 cells were also used, a human monocyte cell line. Culture was carried out in Roswell Park Memorial Institute medium (RPMI) containing 10% FBS and 100 U/ml-100 µg/mL of penicillin-streptomycin.

### MTT assay

Caco-2 cells were treated with different concentrations of *Lg*-EPS (250, 500, 1000 or 2000 µg/mL) for 24 h and 48 h at 37°C in 5% CO_2_. For this, 10^5^ cells/well were placed in 96-well plates (Microplate ELISA; Greiner Bio-one). After incubation with *Lg*-EPS, 10 μL MTT (3-(4,5-dimethylthiazol-2-yl)-2,5-diphenyltetrazolium) bromide (MTT cell proliferation kit; MT01000; OzBiosciences) was added for 4 h. The detergent from the MTT kit (100 μL) was then added overnight at 37°C in order to dissolve the crystals. Absorbance was read at 570 nm in a spectrophotometer. In parallel, a control was performed in the presence of Caco-2 cells only (basal condition) and another control was prepared using medium only.

### Expression of pro-apoptotic genes by Caco-2 cells in the presence of Lg-EPS

Caco-2 cells were cultured for 14 days post-confluence, with the culture medium changed every 2–3 days. For this purpose, 10^6^ Caco-2 cells were seeded into each well of a 12-well plate. *Lg*-EPS were then added at a concentration of 2 mg/mL for 6 hours. The control consisted of untreated Caco-2 cells (basal condition). The cells were washed twice with PBS and then recovered in RA1 buffer to carry out extraction of mRNA, followed by RT-PCR and q-PCR.

### Expression of pro-inflammatory cytokine genes by Caco-2 cells in the presence of L. gasseri

Caco-2 cells were cultured for 14 days after reaching confluence, with the culture medium changed every 2–3 days. For this purpose, 10^6^ Caco-2 cells were seeded into each well of a 12-well plate (Nunclon^TM^ Delta surface; 140,681; Thermo Scientific) for each condition. Subsequently, the Caco-2 cells were co-incubated with *L. gasseri* at different multiplicities of infection (MOI; 1:1, 1:5, and 1:10) for 24 h at 37°C in 5% CO_2_. Caco-2 cells alone were used as the control group (untreated Caco-2 cells; basal condition). The cells were then recovered in RA1 buffer in order to extract mRNA and perform RT-PCR and q-PCR. A control of Caco-2 cells only was prepared in order to standardize and compare the other conditions.

### Expression of pro-inflammatory cytokine genes by Caco-2 cells exposed to 1.5% DSS in the presence of Lg-EPS

Caco-2 cells were cultured for 14 days after reaching confluence, with the culture medium changed every 2–3 days. For this purpose, 10^6^ Caco-2 cells were seeded into each well of a 12-well plate for each condition. Subsequently, the Caco-2 cells were exposed to DMEM containing 1.5% dextran sodium sulfate (DSS) for 24 h in order to induce epithelial lesions and to increase the expression of pro-inflammatory cytokines according to an adapted version of the protocol described by Toutounji et al. [37]. *Lg*-EPS were added at a concentration of 250 µg/mL either during co-incubation for 24 h, or prior to and following exposure for 6 h. The control consisted of untreated Caco-2 cells (basal condition). DSS corresponds to Caco-2 cells exposed to DSS for 24 h (positive control). The cells were washed twice in PBS before recovering them in RA1 buffer to carry out extraction of mRNA, followed by RT-PCR and q-PCR.

### Expression of cytokine genes and receptors by macrophages derived from THP-1 cells in the presence of L. gasseri or Lg-EPS

A suspension of 10^6^ THP-1 cells was differentiated into macrophages by the addition of 100 ng/mL phorbol (phorbol-12-myristate13-acetate; 10,515,491; Thermo Scientific) for 72 h, followed by a rest phase for 24 h in RPMI medium. The macrophages were then incubated for 6 h with lipopolysaccharides (LPS) at a concentration of 250 ng/mL (LPS from *E. coli* O111; L5293; Sigma). In parallel, the macrophages were co-incubated with *L. gasseri* at different MOI (1:1; 1:5 or 1:10) or with *Lg*-EPS at different concentrations (250, 500 or 1000 µg/mL). Untreated macrophages represented the control group (MΦ; basal condition) and macrophages exposed to LPS represented a positive control (LPS). The cells were then recovered in RA1 buffer to carry out mRNA extraction, followed by RT-PCR and q-PCR.

### Purification of mRNA

mRNA was extracted by the NucleoSpin RNA® method (Kit NucleoSpin RNA 250; 740,955.250; Macherey-Nagel). In brief, each sample was lysed directly in an Eppendorf tube using RA1 buffer. The lysate was incorporated in a column with a silica membrane. The content was then transferred and 70% ethanol added. The column was placed in a new tube and membrane desalting buffer was added. DNase was added for 15 min to remove DNA. The column was then washed in RAW2 and RA3 buffer. Finally, mRNA was eluted with RNase-free water and the quantity of mRNA (in μg/μL) of each sample was measured using a spectrophotometer (DeNovix DS-11).

### RNA quality and purity

RNA quality and purity were assessed. The concentration and purity of all RNA samples was evaluated using DeNovix DS-11 spectrophotometer (260/280 nm and 260/230 nm ratios). To assess the quality of the RNA, an RNA 6000 Pico Kit for the 2100 Bioanalyzer system was used (Agilent RNA 6000 Pico Kit; 5067–1513), following the protocol provided with the kit. Briefly, an RNA ladder was reconstituted in Milli-Q water after heat denaturation for 2 min at 95°C. The gel matrix was equilibrated using centrifuge for 10 min at 1500 ×g. The dye was added to the gel matrix at a ratio of 1 µL dye/65 µL gel, followed by centrifugation for 10 min at 13 000 ×g. A total of 9 µL gel-dye mixture was loaded onto the chip at position (C) and pressurization was performed. 9 µL of the gel-dye mixture were dispensed into the wells marked (G) and 9 µL of RNA Pico conditioning solution were added to the well-marked (CS). 5 µL of RNA 6000 Pico Marker were dispensed into the experimental wells, to which 1 µL of RNA or nuclease-free water was added, and the analysis program was initiated. All the corresponding data are available online [35].

### Complementary DNA synthesis with reverse transcriptase

Synthesis of DNA was carried out using high-capacity cDNA reverse transcription (RT) master mix (4368813; Applied Biosystems). Briefly, the mix contained 2 μL 10X RT buffer, 2 μL 10X RT random primers, 0.8 μL 25X dNTP mix, 1 μL reverse transcriptase, and 4.2 μL RNase-free water, in order to obtain a final volume of 10 μL/sample. In order to obtain samples with 1000 ng mRNA/cDNA in a volume of 10 μL, RNase-free water was added to the sample. The whole sample was placed in a thermocycler (Bio-RAD, iCycler) to obtain cDNA after several cycles (10 min at 25°C, 2 h at 37°C and 5 min at 85°C). The samples were then stored at 4°C.

### Q-PCR

PCR was carried out in 96-well microtitre plates (MicroAmp Fast Optical 96 well; Applied Biosystems) specific to the StepOne machine. For each sample, a mix was prepared with 0.25 μL sense and anti-sense primers (**Supplementary Table S1**), 5 μL Sybr Green (4385612; Applied Biosystems), 2.5 μL RNase-free water, and 2 μL cDNA (diluted 1/5), to a total volume of 10 μL/well. Once the deposits had been made, the plate was inserted into the StepOne machine and the programme started (95°C for 20 s; 40 cycles at 95°C for 3 s and 60°C for 30 s; 95°C for 15 s; 60°C for 1 min; and 95°C for 15 s). Relative gene expression was calculated using the ΔΔCt method. Briefly, relative mRNA expression levels were normalized to the housekeeping gene GAPDH and the results were expressed as fold change relative to control conditions (untreated cells).

### Reporter assays of AhR induction

AhR reporter activity was measured using the HT29-Lucia^TM^ AhR cell line (ht2l-ahr; InvivoGen), a commercially available system in which a luciferase reporter gene is stably integrated under the control of AhR-responsive elements. This system allows direct quantification of AhR transcriptional activity without the need for empty vector controls. Cells were treated as described below and reporter activity was measured according to the manufacturer’s instructions and normalized to control conditions. Briefly, a suspension of 2.8 × 10^5^/ml HT29-Lucia^TM^ AhR cells was resuspended in test medium (DMEM containing 10% FBS, 100 U/ml-100 µg/mL penicillin-streptomycin, without Normocin^TM^ and Zeocin^TM^). The cell suspension (180 µl; ~50 000 cells per well) was then added to a flat-bottom 96-well plate (F96 Microwell plate; 236,108; NUNC). The sample (20 µL; EPS at 250, 500 or 1000 µg/ml) was then added to each well, including FICZ at 5 µg/mL (positive control or AhR ligand; tlrl-ficz; InvivoGen) or CH-223191 at 5 µg/mL (negative control or AhR inhibitor; inh-ch22; InvivoGen). The plate was incubated at 37°C in a CO_2_ incubator for 24 h. QUANTI-Luc^TM^ 4 reagent working solution was prepared (Luminescence detection kit; rep-qlc4lg1; InvivoGen) and 20 µL of HT29-Lucia^TM^ AhR stimulated cell supernatant was transferred into a 96-well white (opaque) or black plate. QUANTI-Luc^TM^ 4 reagent working solution (50 µL) was then added. Luminescence in the wells was measured immediately in a Fluostar Omega (BMG Labtech) and the results expressed as “Fold change” relative to the control condition (HT29-Lucia^TM^ AhR cells alone).

### Determination of IL-6 and IL-10 cytokine levels secreted by macrophages

A suspension of 10^6^ THP-1 cells was differentiated into macrophages by adding 100 ng/mL phorbol for 72 h followed by a rest phase for 24 h in RPMI medium. The cells were then incubated for 16 h in the presence of LPS at a concentration of 250 ng/mL as well as *L. gasseri* or *Lg*-EPS (MOI 1:10, or at a concentration of 1 mg/mL, respectively: optimal condition observed during expression experiments). After 16 h, the supernatants were recovered. The concentration of IL-6 (Invitrogen^TM^ Human IL-6 ELISA eBioscience^TM^ kit; 15,551,037; Thermo Scientific) and IL-10 (Invitrogen^TM^ Human IL-10 ELISA eBioscience^TM^ kit; 15,551,047; Thermo Scientific) in the cell supernatants was measured by ELISA, as described by the manufacturer. The control group consisted of untreated macrophages (MΦ; basal condition) and the positive control was macrophages exposed to LPS (LPS). The concentrations of IL-6 and IL-10 were then estimated from standard curves provided with the kits and the results expressed as “Fold Release” by normalizing with the positive control.

### Fungal cultures

The fungal strain used was *C. albicans* the standard reference strain SC5314 (wild-type) used worldwide to study the biology, genetics, and pathogenic mechanisms of *C. albicans*. Fungal culture was carried out in Sabouraud (Sabouraud dextrose broth; 1.08339.0500, Millipore), with shaking for 18 h at 37°C. The culture obtained was then centrifuged at 2500 rpm for 5 min and washed twice in PBS.

### Effect of L. gasseri and Lg-EPS on C. albicans biofilms

A suspension of 10^5^ yeasts was placed in a 96-well microtitre plate (Microtest plate 96-well; Sarstedt) containing 200 µL RPMI 1640 medium in the presence of *L. gasseri* (MOI: 1:1 or 1:10) or *Lg*-EPS (1 or 2 mg/mL) at a temperature of 37°C for 24 h. The wells were then washed twice in PBS before adding 200 µL 0.5% crystal violet for 5 min (32909-250 ML; Honeywell Fluka). The wells were then washed twice in Milli-Q water before eluting with acetic acid (33%) for 20 min. Finally, the solution was transferred to a new plate before reading the absorbance at 595 nm (Fluostar Omega, BMG Labtech). Three controls were prepared: an untreated culture of *C. albicans* (basal condition) and a culture of *C. albicans* exposed to 100 µg/mL caspofungin (50 mg; OHRE Pharma; positive control) or 100 µg/mL fluconazole (Fluconazole Kabi® 2 mg/mL; Fresenius Kabi; positive control).

### Effect of Lg-EPS on C. albicans growth

A suspension containing 10^3^ yeasts was placed in a 15 mL flask (polypropylene conical tube; 352,196; Falcon) in the presence of EPS at a concentration of 250, 500, 1000, or 2000 µg/mL, and in a final volume of 2 mL Sabouraud broth at 37°C. Fungal growth was then followed over 8 h, using 100 µL of each sample, by measuring the absorbance at 0, 4, 6 and 8 h at 620 nm (Fluostar Omega, BMG Labtech). Absorbance at 620 nm was measured and corrected by subtracting the blank (i.e. Sabouraud culture medium alone). Fungal growth was quantified by subtracting the baseline absorbance at T0 from the corresponding values at T4, T6, and T8 for each condition. The resulting values represent OD620 and were used as a proxy for fungal growth over time. Two controls were prepared: an untreated culture of *C. albicans* (basal condition) and a culture of *C. albicans* exposed to 100 µg/mL caspofungin (positive control).

### Adhesion of C. albicans to human intestinal epithelial Caco-2 cells treated with EPS

Caco-2 cells were maintained for 14 days after reaching confluence, with the culture medium changed every 2–3 days. For this purpose, 10^5^ Caco-2 cells were added to each well of a 96-well plate (655083, Greiner Bio-One). Subsequently, 5.10^5^ cells of *C. albicans* previously labeled with calcein AM (65–0853-81, Invitrogen) were added to each well (the control was untreated yeasts). At the same time, *Lg*-EPS were added to the wells in the test condition at a concentration of 1 or 2 mg/mL. A negative control containing caspofungin at a concentration of 100 µg/mL was also prepared. After 2 h of incubation and two washes with PBS, fluorescence was measured using a FLUOstar Omega machine (BMG Labtech).

### Filamentation of C. albicans treated with EPS

A total of 10^5^ yeasts were seeded into each well of a 12-well plates (2 mL final volume) at 37°C (the control was untreated yeasts). At the same time, *Lg*-EPS were added to the wells in the test condition at a concentration of 1 or 2 mg/mL. A negative control containing caspofungin at a concentration of 100 µg/mL was also prepared. Cells were monitored for 12 h using a Leica DMI8 inverted microscope (20x magnification).

### Confocal microscopy of co-incubation between L. gasseri and C. albicans

*C. albicans* SC5314 was grown in Sabouraud broth and *L. gasseri* was grown in MRS broth. The cultures were centrifuged at 800 g for 5 min and washed with PBS. The bacteria were then labeled with 200 µL DAPI solution (1 µg/mL, 1/1000; Thermo Scientific) for 30 min at 37°C. A total of 200 µL rhodamine-concanavalin A solution (ConA-Rhod, 0.1 µg/µL, 1/200, 25 mg; Vector) was added to label *C. albicans* mannans for 30 min at 37°C. Both sets of cells were then washed in PBS. Yeasts and bacteria were co-incubated at a final concentration of 5 × 10^5^ yeasts and 5 × 10^6^ bacteria (MOI 1:10) by depositing 100 µL of inoculum on a slide and incubating at 37°C for 1 h. At the end of incubation, the cells were fixed with 100 µL 3.7% (v/v) formaldehyde solution for 1 h. The supernatant was removed and the cells were washed three times in PBS. Finally, Eukitt was added (EUKITT Classic 100 mL; Dutscher) to mount the cells between the slides and the coverslip, and the sample was conserved until confocal microscopy was performed (LSM 710 AiryScan, Zeiss).

### Statistical analyses

The data are presented as the mean ± standard deviation (SD) or median and interquartile range [IQR; Q1–Q3]. Statistical comparisons were carried out using the non-parametric Mann-Whitney test or ANOVA. Graphical data are shown as median or mean. All statistical analyses were carried out using GraphPad Prism® 10.0 (GraphPad, San Diego, CA, USA). *p* ≤ 0.05 was considered statistically significant.

## Results

### Characterization of the monosaccharide composition of Lg-EPS and their effects on cell proliferation and the expression of pro-apoptotic markers by Caco-2 cells

**Figure 1.**
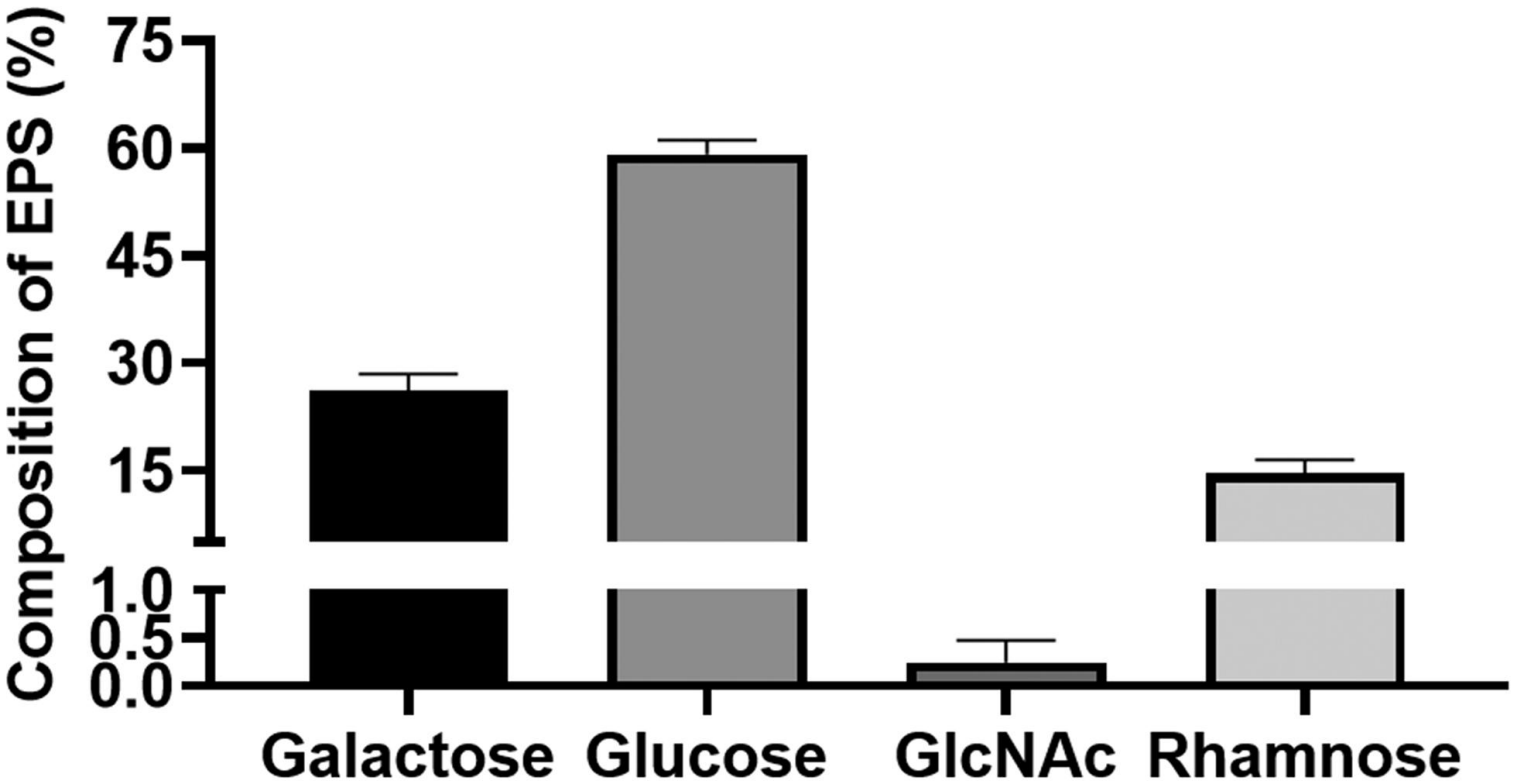
Representative data on *Lg*-EPS monosaccharide composition (%) from GC-FID analyses. The results were obtained from several EPS samples (*n* = 18 batches).

The monosaccharide composition of *Lg*-EPS was determined using trimethylsilyl (TMS) derivatives, and the monosaccharides were characterized and quantified by GC/FID based on the retention times of their isomers (Figure 1). EPS contained significant proportions of sugars: glucose (59.1%), galactose (26.0%) and rhamnose (14.6%), and a small quantity of N-acetyl-glucosamine (0.3%). Assessment of the purity of *Lg*-EPS by SDS-PAGE, Western blot and spectrophotometry indicated that major microbial contaminants were absent or present only in trace amounts, close to the detection limits of the methods used (**Supplementary Fig. S1A – C**). *Lg*-EPS significantly decreased cell proliferation after 24 h (Ordinary One-way ANOVA; F (4, 62) = 9.387; *p* < 0.0001) and 48 h (Kruskal-Wallis test; H = 25.83; *p* < 0.0001) (**Supplementary Fig. S2A-B**), but only at a concentration ≥ 2 mg/mL when compared to the control (Caco-2 cells alone). In parallel, the expression of pro-apoptotic markers such as BAX (Mann-Whitney test; U = 2; *p* < 0.0001), caspase 1 (Mann-Whitney test; U = 56; *p* = 0.0118) and caspase 9 (Mann-Whitney test; U = 34; *p* = 0.0004) decreased significantly when the cells were exposed to 2 mg EPS (**Supplementary Fig. S2C-E**).

### Effect of L. gasseri and Lg-EPS on Caco-2 cells in the presence of DSS

**Figure 2.**
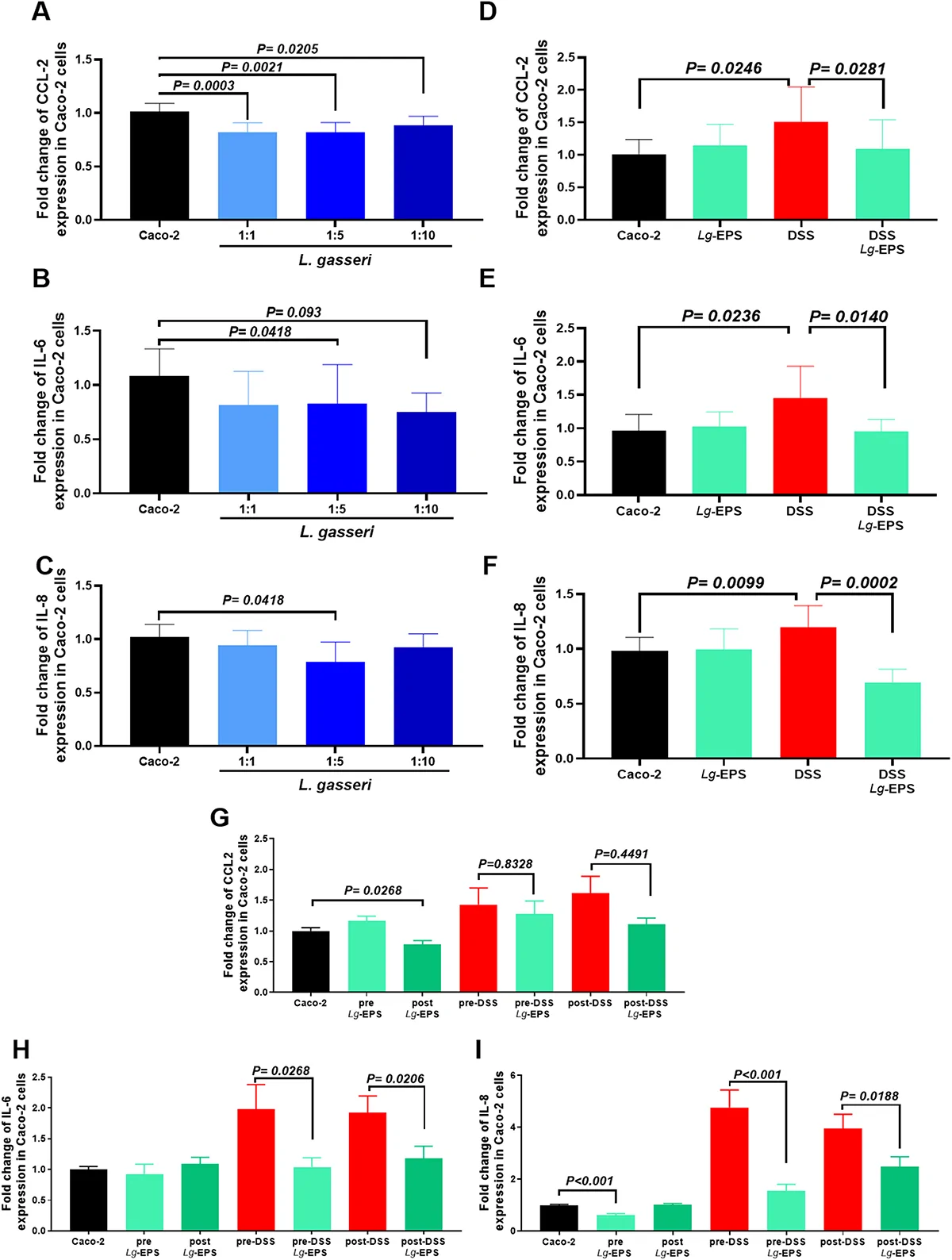
Activity of *L. gasseri* or *Lg*-EPS on the modulation of pro-inflammatory cytokine/chemokine expression in Caco-2 cells. (A–C) Relative mRNA expression levels of CCL2 (A), IL-6 (B) and IL-8 (C) in Caco-2 cells. Caco-2 corresponds to the control group (Caco‑2 cells alone); 1:1 corresponds to Caco-2 cells co-incubated with *L. gasseri* at a MOI of 1:1; 1:5 corresponds to Caco-2 cells co-incubated with *L. gasseri* at a MOI of 1:5; 1:10 corresponds to Caco-2 cells co-incubated with *L. gasseri* at a MOI of 1:10. (D–F) Relative mRNA expression levels of CCL2 (D), IL-6 (E) and IL-8 (F) in Caco-2 cells exposed to DSS (1.5%). Caco-2 corresponds to the control group (untreated Caco-2 cells); *Lg*-EPS corresponds to Caco-2 cells co-incubated with EPS (250 µg/mL); DSS corresponds to Caco-2 cells stimulated with DSS; DSS + *Lg*-EPS corresponds to Caco-2 cells exposed to DSS and co-incubated with EPS (250 µg/mL). (G-I) Relative mRNA expression levels of CCL2 (G), IL-6 (H) and IL-8 (I) in Caco-2 cells exposed to DSS (1.5%) for 24 h and pre- or post-treated with *Lg*-EPS for 6 h. Caco-2 corresponds to the control group (untreated Caco-2 cells, basal condition); pre-*Lg*-EPS or post-*Lg*-EPS corresponds to Caco-2 cells pre- or post-incubated with EPS (250 µg/mL) for 6 h; pre-DSS corresponds to Caco-2 cells exposed to DSS for 24 h and then to DMEM medium for 6 h; pre-DSS *Lg*-EPS corresponds to Caco-2 cells exposed to DSS for 24 h and treated with EPS (250 µg/mL) for 6 h; post-DSS corresponds to Caco-2 cells in DMEM medium for 6 h and exposed to DSS for 24 h; post-DSS *Lg*-EPS corresponds to Caco-2 cells treated with EPS (250 µg/mL) for 6 h and exposed to DSS for 24 h. Data represent three (A-F) or four (G-I) independent biological replicates per condition, each performed in triplicate.

The expression of cytokines IL-6, IL-8 and CCL2 by Caco-2 cells was measured at different *L. gasseri* MOI, ranging from 1:1 to 1:10. Pro-inflammatory cytokine and chemokine expression showed only modest changes, although these decreases were significant in the presence of *L. gasseri* at a MOI of 1:1 (Mann-Whitney test; CCL2: U = 1; *p* = 0.0003), 1:5 (Mann-Whitney test; IL-6: U = 12; *p* = 0.0418; IL-8: U = 12; *p* = 0.0418; CCL2: U = 4; *p* = 0.0021) or 1:10 (Mann-Whitney test; IL-6: U = 6; *p* = 0.0093; CCL2: U = 8; *p* = 0.0205) (Figure 2(A–C)). The expression of these cytokines was then assessed in the presence of various *Lg*-EPS concentrations (ranging from 250–1000 µg/mL). A model of Caco-2 cells exposed to DSS (1.5%) was used to evaluate the possible anti-inflammatory effects of these EPS. Exposure to DSS provided a model of inflammation similar to that found in ulcerative colitis. Pro-inflammatory cytokine and chemokine expression increased significantly in the presence of DSS (Mann-Whitney test; IL-6: U = 17; *p* = 0.0236; IL-8: U = 17; *p* = 0.0099; CCL2: U = 21; *p* = 0.0246) (Figure 2(D–F)). Cells treated concomitantly with DSS and *Lg*-EPS, notably at a concentration of 250 µg/mL, significantly decreased the expression of these pro-inflammatory mediators when compared to cells treated with DSS alone (Mann-Whitney test; IL-6: U = 7; *p* = 0.0140; IL-8: U = 0; *p* = 0.0002; CCL2: U = 11; *p* = 0.0281) (Figure 2(D–F)). Similar effects were observed at a concentration of 500 µg/mL (**data not shown**). To investigate further, the cells were pre- and post-treated with *Lg*-EPS after challenge with DSS (1.5%). The results show that *Lg*-EPS retained its immunomodulatory effects by decreasing the expression of the pro-inflammatory cytokines IL-6 (Mann-Whitney test; U = 30; *p* = 0.0268 and U = 25; *p* = 0.0206) (Figure 2(G)) and IL-8 (Mann-Whitney test; U = 6; *p* < 0.0001 and U = 28; *p* = 0.0188) (Figure 2(H)). Similarly, the expression of the cytokine CCL2 decreases, but not significantly (Figure 2(I)).

### Effect of L. gasseri and Lg-EPS on macrophages stimulated by LPS

**Figure 3.**
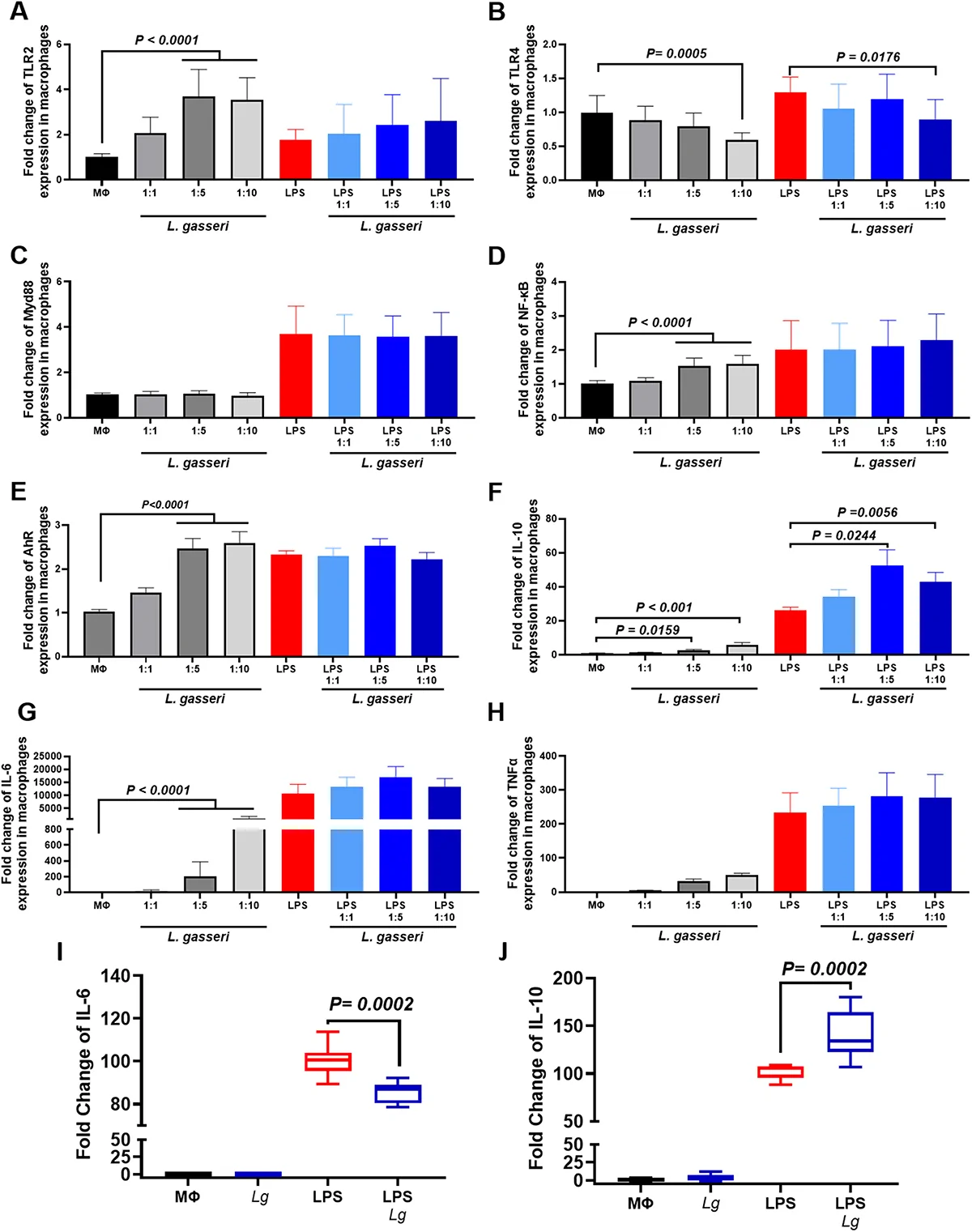
Effect of *L. gasseri* on the expression of inflammatory receptors or mediators of macrophages and on levels of IL-6 and IL-10 secreted by macrophages exposed to LPS. (A–H) Relative mRNA expression levels of TLR2 (A), TLR4 (B), Myd88 (C), NF-κB (D), AhR (E), IL-10 (F), IL-6 (G) and TNFα (H), respectively, in macrophages. MΦ represents the control group (macrophages alone); 1:1, 1:5 and 1:10 correspond to macrophages co-incubated with *L. gasseri* at different MOI. LPS represents the positive control (macrophages exposed to LPS). LPS 1:1, LPS 1:5 and LPS 1:10 correspond to macrophages exposed to LPS and co-incubated with *L. gasseri* at different MOI. (I-J) Release of IL-6 (I) and IL-10 (J) by macrophages co-incubated with bacteria at a MOI of 1:10. MΦ represents a control group (macrophages alone); *L. gasseri* corresponds to macrophages co-incubated with *L. gasseri*; LPS represents a positive control (macrophages exposed to LPS); LPS + *L. gasseri* corresponds to macrophages exposed to LPS and co-incubated with *L. gasseri*. Data represent three independent biological replicates per condition, each performed in triplicate.

The effect of *L. gasseri* was investigated in macrophages stimulated with LPS for 6 h (derived from THP-1 cells) to induce the increased expression of cytokines, mediators, and receptors involved in the inflammatory signaling pathway, in order to study the anti-inflammatory effects of this bacterium (Figure 3(A–H)). Co-incubation with *L. gasseri* (MOI 1:10) significantly decreased TLR4 expression (Mann-Whitney test; U = 7; *p* = 0.0176 and U = 6; *p* = 0.0005) and increased IL-10 expression (Mann-Whitney test; U = 10; *p* = 0.0056 and U = 1; *p* < 0.001) compared with both LPS-exposed macrophages and untreated control macrophages (Figure 3(B,F)). In parallel, the addition of *L. gasseri* (MOI 1:5 and 1:10) significantly increased the expression of TLR2 (Mann-Whitney test; U = 0; *p* < 0.0001) (Figure 3(A)), NF-κB (Mann-Whitney test; U = 0; *p* < 0.0001) (Figure 3(D)), AhR (Mann-Whitney test; U = 0; *p* < 0.0001) (Figure 3(E)) and cytokines IL-6 (Mann-Whitney test; U = 0; *p* < 0.0001) and IL-10 (Mann-Whitney test; U = 18; *p* = 0.0159 and U = 1; *p* < 0.001) (Figure 3(F,G)) when compared to macrophages alone.

**Figure 4.**
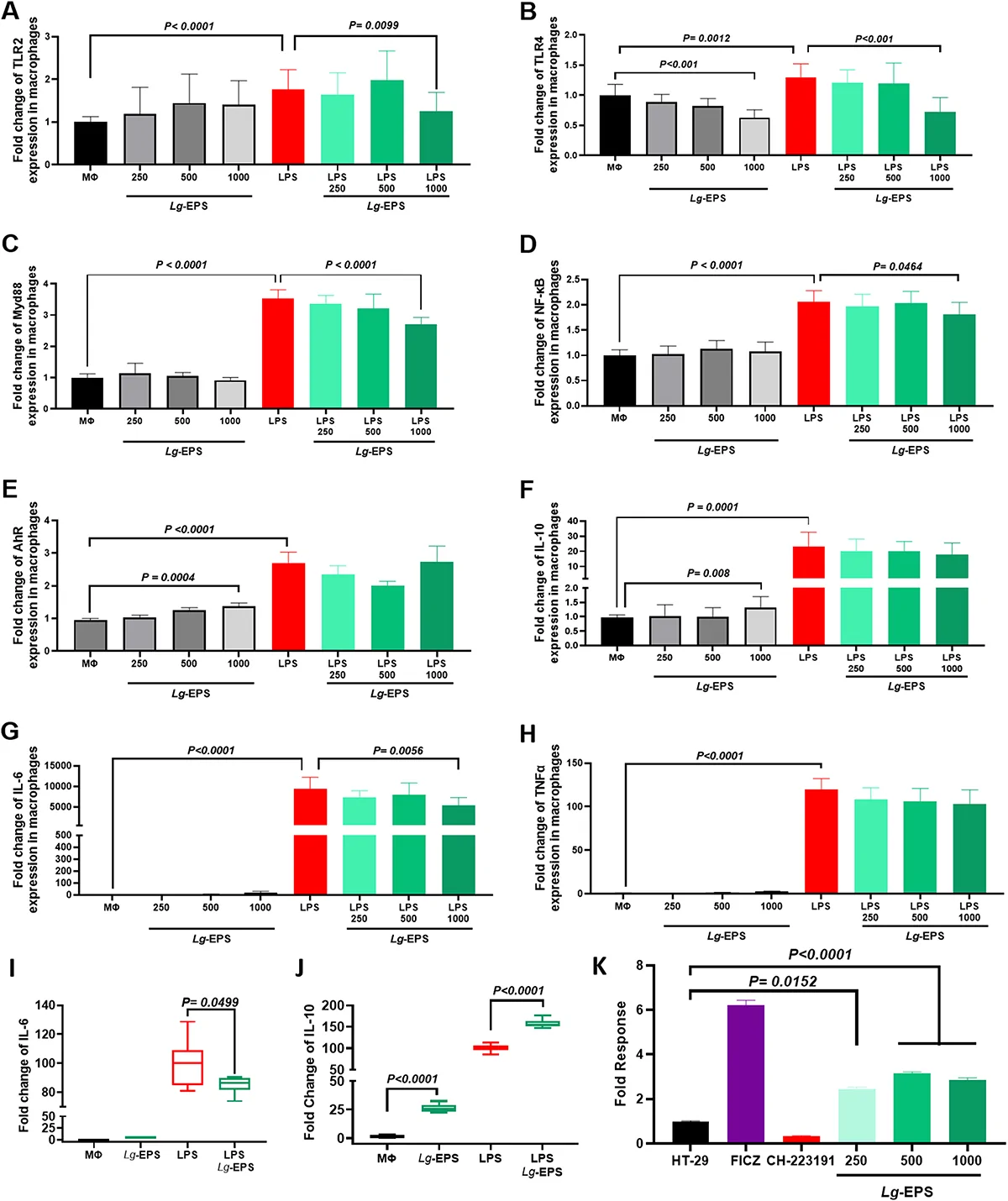
Effect of *Lg*-EPS on the expression of inflammatory receptors or mediators of macrophages, on levels of IL-6 or IL-10 secreted by macrophages exposed to LPS, and induction of AhR activity by *Lg*-EPS in HT29-Lucia™ AhR cells. (A–H) Relative mRNA expression levels of TLR2 (A), TLR4 (B), Myd88 (C), NF-κB (D), AhR (E), IL-10 (F), IL-6 (G) and TNFα (H), respectively, in macrophages. MΦ represents a control group (macrophages alone); 250, 500 and 1000 correspond to macrophages treated with *Lg*-EPS at 250, 500 or 1000 µg/mL, respectively. LPS represents a positive control (macrophages exposed to LPS). LPS 250, LPS 500 or LPS 1000 correspond to macrophages exposed to LPS and treated with *Lg*-EPS at 250, 500 or 1000 µg/mL, respectively. (I-J) Release of IL-6 (I) and IL-10 (J) by macrophages exposed to *Lg*-EPS at 1 mg/mL. MΦ represents a control group (macrophages alone); *Lg*-EPS corresponds to macrophages treated with EPS. LPS represents a positive control (macrophages exposed to LPS); LPS + *Lg*-EPS corresponds to macrophages exposed to LPS and treated with EPS. (K) Induction of AhR activity by *Lg*-EPS in HT29-Lucia™ AhR cells. HT29 represents a control group (cells alone); FICZ represents a positive control (AhR ligand; 5 µg/mL); CH-223191 represents a negative control (AhR inhibitor; 5 µg/mL); 250, 500 and 1000 correspond to macrophages treated with *Lg*-EPS at 250, 500 or 1000 µg/mL, respectively. Data represent three independent biological replicates per condition. Relative mRNA expression levels and cytokine release by macrophages (A-J) were assessed in triplicate, whereas induction of AhR activity in HT29-Lucia^TM^ AhR cells (K) was assessed in sextuplicate.

The TLR4 receptor also decreased significantly when *Lg*-EPS (1 mg/mL) were added to macrophages (Mann-Whitney test; U = 8; *p* < 0.001) (Figure 4(B)). Treatment of the macrophages with LPS led to a significant increase (Mann-Whitney test; U = 0; *p* < 0.0001) in the expression of TLRs, mediators of transcription factors MyD88 and NF-κB, AhR and cytokines IL-6, IL-10 and TNFα (Figure 4(A–H)). The addition of *Lg*-EPS (1 mg/mL) significantly reduced the increase in expression of TLRs (Mann-Whitney test; TLR2 U = 12; *p* = 0.0099; TLR4 U = 1; *p* < 0.001), MyD88 (Mann-Whitney test; U = 0; *p* < 0.0001), NF-Κb (Mann-Whitney test; U = 15; *p* = 0.0464) and IL-6 (Mann-Whitney test; U = 10; *p* = 0.0056) compared to macrophages treated with LPS alone (Figure 4(A–E) and Figure 4(G)). Concerning AhR and cytokine IL-10, *Lg*-EPS (1 mg/mL) induced a non-significant up-regulation compared to macrophages treated with LPS, but significantly increase both markers (Mann-Whitney test; U = 13; *p* = 0.0004 and U = 26; *p* = 0.008, respectively) relative to macrophages alone (Figure 4(E,F)).

Following our previous observations, macrophages were treated simultaneously with LPS and *L. gasseri* (MOI 1:10) or EPS (1 mg) for 16 h. Cytokine secretion was then measured (Figure 3(I,J) and Figure 4(I,J)). LPS significantly increased IL-6 (Mann-Whitney test; U = 0; *p* < 0.0001) and IL-10 secretion (Mann-Whitney test; U = 0; *p* < 0.0001). However, in LPS-stimulated macrophages, *L. gasseri* or EPS decreased IL-6 (Mann-Whitney test; U = 2; *p* = 0.0002 and U = 13; *p* = 0.0499) and increased IL-10 secretion (Mann-Whitney test; U = 2; *p* = 0.0002 and U = 0; *p* < 0.0001) compared to the positive control (MΦLPS) (Figure 3(I,J) and Figure 4(I,J)). *Lg*-EPS also increased IL-10 levels relative to macrophages alone (MΦ) (Mann-Whitney test; U = 0; *p* < 0.0001) (Figure 4(J)).

In a second step, the qPCR results on the relative expression of AhR in macrophages (Figure 4(E)) were verified using AhR reporter cells known as HT29-LuciaTM AhR (Figure 4(K)). The results showed that cells treated with the FICZ ligand had a 6.2-fold increase in AhR response, while cells exposed to the CH-223191 inhibitor exhibited a 0.34-fold decrease compared to unexposed cells. The addition of *Lg-*EPS at various concentrations, ranging from 0.25 to 1 mg/mL, resulted in an increase in response to 2.5, 3.1 and 2.9-fold change, respectively. These results highlight an increase in induction of AhR activity by *Lg-*EPS in HT29-Lucia™ AhR cells (Kruskal-Wallis test; H = 108.9; *p* < 0.0001).

### Interaction with and inhibitory activities of L. gasseri and Lg-EPS on C. albicans

**Figure 5.**
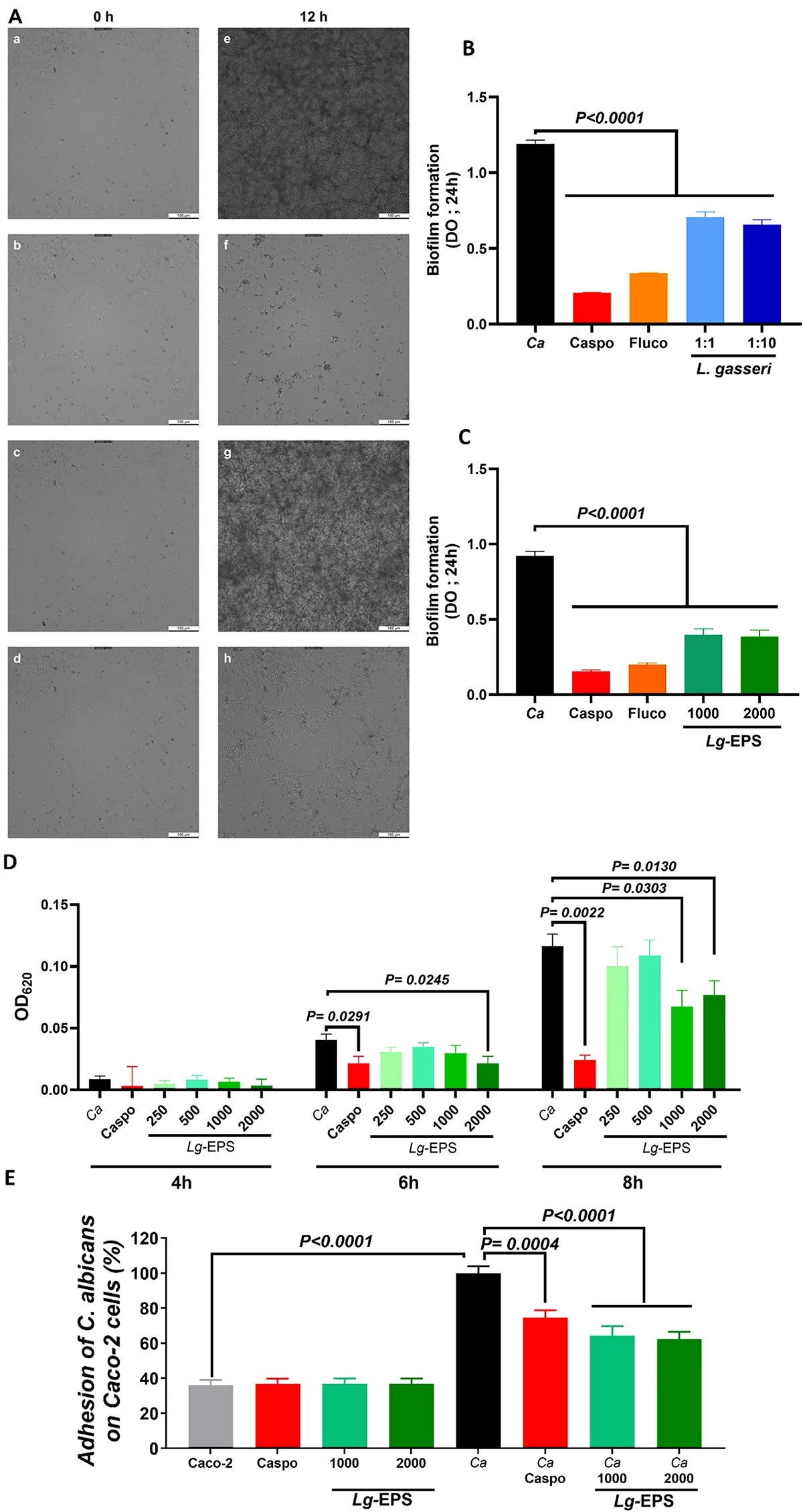
Inhibitory activities of *L. gasseri* and *Lg*-EPS on *C. albicans*. (A) Effects of *Lg-*EPS on *C. albicans* filamentation after 0 h and 12 h. Filamentation of *C. albicans* alone after 0 h (a) or 12 h (e), with caspofungin after 0 h (b) or 12 h (f), with *Lg*-EPS (1 mg) after 0 h (c) or 12 h (g) and with *Lg*-EPS (2 mg) after 0 h (d) or 12 h (h). The scale bar corresponds to 100 µm. Data are representative of three independent experiments. (B-C) Effects of *L. gasseri* or *Lg*-EPS on biofilm formation by *C. albicans*. Biofilm formation by *C. albicans* strain SC5314 at 37°C under standard conditions or after co-incubation with *L. gasseri* (B) or *Lg-*EPS (C) for 24 h. (D) Effects of *Lg-*EPS on *C. albicans* growth after 4, 6 and 8 h of co-incubation. (E) Effects of *Lg-*EPS on fungal adhesion to Caco-2 cells after 2 h exposure. *ca* represents the control group (*C. albicans* alone); Caspo represents the negative control (*C. albicans* treated with caspofungin at 100 µg/mL); Fluco represents the second negative control (*C. albicans* treated with fluconazole at 100 µg/mL); *Lg-*EPS corresponds to *C. albicans* treated with *Lg*-EPS. Data represent three independent biological replicates per condition. Biofilm formation by *C. albicans* (B-C) were assessed in octuplicate, whereas fungal growth (D) were assessed in duplicate, and fungal adhesion (E) were assessed in sextuplicate.

*C. albicans* was co-incubated with *L. gasseri* (MOI 1:10) for 2 h and then observed by confocal microscopy **(Supplementary Figure S3A-C**). Confocal microscopy showed that *L. gasseri* and *C. albicans* were observed in close spatial proximity during co-incubation, without clear spatial segregation. However, these observations did not allow us to confirm direct interactions between the two species (**Supplementary Figure S3C1-C3**). For *C. albicans* filamentation, the addition of caspofungin resulted in the absence of filamentation (Figure 5(ab) and 5(Af)). Exposure of *C. albicans* to *Lg*-EPS (2 mg/mL) (Figure 5(Ad) and 5(Ah)), also showed a change in filamentation, which decreased compared to the control (untreated *C. albicans*; Figure 5(Aa) and 5(Ae)). However, no significant variation in yeast filamentation was observed in the presence of *Lg*-EPS at 1 mg (Figure 5(Ac) and 5(Ag)), compared with the control (Figure 5(Aa) and 5(Ae)). Studies on biofilm formation showed that co-incubation of *C. albicans* with *L. gasseri*, and with *Lg*-EPS, led to a significant decrease (Mann-Whitney test; U = 0; *p* < 0.0001) in biofilm formation by the yeast (Figure 5(B,C)). Co-incubation with *Lg*-EPS decreased the fungal growth after 6 h at a concentration of 2 mg/mL (Mann-Whitney test; U = 7; *p* = 0.0245) and after 8 h both concentration of 1 and 2 mg/mL resulted in reduction of fungal growth (Mann-Whitney test; U = 4.5; *p* = 0.0303 and U = 3; *p* = 0.0130) (Figure 5(D)). Alongside of these results, the addition of *Lg*-EPS (1 or 2 mg/mL) decreases fungal adhesion to Caco-2 cells after 2 h of exposure (Mann-Whitney test; U = 22; *p* < 0.0001 and U = 15; *p* < 0.0001, respectively) (Figure 5(E)).

## Discussion

IBDs are characterized by genetic predisposition, and environmental and immunological factors, which influence the intestinal microbiota composition [38–40]. This microbiota, subject to change by a number of factors, including diet, stress, genetics, medication, and age, plays a crucial role in IBDs [41–43]. Patients with IBD have a decrease in intestinal microbiota biodiversity, particularly of anaerobic species of the phyla *Firmicutes* [44]. This decrease favors the proliferation of opportunistic pathogens such as *C. albicans*, to the detriment of some protective bacteria, such as *Lactobacillus* spp., which play a role in the prevention of intestinal inflammation [20,42] and in the reinforcement of tight junctions [45,46]. Studies have reported that these lactic acid producing bacteria (LAB) have beneficial and protective activity against DSS-induced colitis by decreasing the burden of harmful bacteria like *Shigella* or *Turicibacter* [5] or colonization with opportunistic pathogens like *Candida* spp [31]. These bacterial species can also synthesize microbial metabolites, such as short-chain fatty acids (SCFAs) produced by bacterial fermentation, which participate in the maintenance of intestinal homeostasis [47,48]. SCFAs are also involved in the inhibition of growth and virulence factors of *Candida* spp [49–51]. The protective effects of LAB also occur through their chitinase activities, which inhibit fungal growth, notably by inducing degradation of the inner part of the fungal cell wall, or by organic acids [31,52]. Thus, these bacteria have the capacity to interact with *Candida* in various ways. However, the role of molecular determinants such as EPS has not yet been characterized.

The results of the present study show that *Lg*-EPS consist essentially of glucose (59.1%), galactose (26.0%) and rhamnose (14.6%), and a very small quantity of N-acetyl-glucosamine (0.3%). *Lg*-EPS were found to decrease the proliferation of Caco-2 cells at a concentration ≥ 2 mg/mL.

The current study also showed that the expression of the pro-apoptotic markers, BAX and caspase 1 and 9, decreased significantly when Caco-2 cells were exposed to *Lg*-EPS at 2 mg/mL. These data are in agreement with a study demonstrating that EPS from two strains of *L. gasseri* (G10 and H15) were capable of inhibiting the proliferation of a human cervical cancer cell line. The authors showed that EPS from strain H15 did not activate BAX or caspase 3, in contrast to EPS from strain G10 [9]. Taken together, these results indicate that the inhibition of proliferation is strain-dependent and may involve distinct mechanisms of action, warranting further investigation.

*L. gasseri* strain LLG1 also significantly reduced the expression of pro-inflammatory cytokines/chemokines IL-6, IL-8, and CCL2, expressed in Caco-2 cells. *Lg*-EPS had comparable anti-inflammatory properties in our Caco-2 cell model. Caco-2 cells exposed to DSS increased the expression of IL-6, IL-8, and CCL2, with a significant decrease in expression when *Lg*-EPS were added. These findings are consistent with previous studies involving porcine intestinal epithelial (PIE) cells. In the first study, a strain of *L. delbrueckii* and its derived EPS were shown to attenuate the pro-inflammatory response in PIE cells following exposure to an enterotoxigenic strain of *E. coli* (ETEC) [53]. In the second study, using the same PIE cell line stimulated with ETEC, acidic fractions of EPS from *L. plantarum* also decreased the production of IL-6, IL-8 and CCL2 *via* negative regulators of receptor TLR4 [54].

In the current study, *L. gasseri* strain LLG1 significantly upregulated the expression and production of the anti-inflammatory cytokine IL-10 in LPS-stimulated macrophages, while levels of the pro-inflammatory cytokine IL-6 were significantly reduced under the same conditions. These results were linked with a significant decrease in expression of receptor TLR4 in the same model. These findings agree with those of Su Oh et al., where probiotics isolated from the stools of neonates, particularly *L. gasseri* strain 4M13, had important anti-inflammatory activity through inhibition of the secretion of several pro-inflammatory cytokines, including IL-6, by murine macrophages (RAW 264.7), during treatment with LPS [10]. In another study, *L. fermentum* IM-12, isolated from human feces, also had immunomodulatory properties because it strongly inhibited the activation of two important cellular signaling pathways, NF-κB and STAT3, and IL-6 secretion in murine peritoneal macrophages stimulated with LPS [55]. Other data obtained from a murine model of DSS-induced colitis demonstrated similar anti-inflammatory properties for three other species: *L. acidophilus*, *L. helveticus* and *L. plantarum*, notably lower IL-6 levels and higher IL-10 levels in the colon were observed in mice treated with different strains [20]. Our findings concerning an increase in mRNA expression and protein level of IL-10, and a decrease in IL-6 and TNFα within macrophages, can be correlated with those of the AhR receptor (Aryl hydrocarbon receptor). This receptor, expressed in immune and nonimmune cells of the intestine, is known to be an immune regulator playing an essential role in the inflammatory response. Indeed, one study has observed the role of this receptor in IL-10 production. More specifically, in agreement with our results, uses AhR-/- mice and murine macrophages (RAW 264.7) over-expressing this receptor to demonstrate its involvement in IL-10 expression via the “AhR-Src-STAT3-IL10” signaling pathway [56]. A second study showed that the AhR receptor is capable of strongly influencing the regeneration of intestinal epithelial cells after inflammatory damage, by maintaining homeostasis and the intestinal barrier integrity [57]. It is well established that activation of the AhR receptor under inflammatory conditions leads to a reduction in pro-inflammatory cytokines such as IL-6, IL-12, or TNFα, while at the same time allowing an increase in regulatory mechanisms involving the anti-inflammatory cytokine IL-10, Foxp3 or the production of antimicrobial peptides [58]. Together, these results suggest that treatment with a probiotic strain such as *L. gasseri* LLG1 is a promising strategy to decrease intestinal inflammation associated with IBDs.

Similar to whole *L. gasseri* cells, the addition of *Lg*-EPS led to a significant decrease in expression of TLRs, MyD88, transcription factor NF-κB and pro-inflammatory cytokine IL-6 in macrophages exposed to LPS. These results were confirmed by our study, which showed that IL-10 levels increased while IL-6 levels decreased in macrophages stimulated with LPS and co-incubated with *Lg*-EPS. These findings are consistent with those reported by Wu et al., who demonstrated that exposure of macrophages to EPS derived from *Bifidobacterium longum*, another LAB, led to increased secretion of the anti-inflammatory cytokine IL-10 [59]. These data also support those obtained by Zhang et al. demonstrated that EPS derived from various *Lactobacillus* strains (*L. plantarum, L. helveticus, L. rhamnosus, L. fermentum* and *L. salivarius*) were strongly able to modulate the immune system. To demonstrate this, the authors used EPS from these different strains on murine macrophages cell line stimulated by LPS and measured the levels of TNFα and IL-10. The anti-inflammatory effects were then measured in a murine model of DSS-induced colitis, where differences were observed according to the species or structural diversity of the EPS on the reduction of pro-inflammatory cytokines IL-6, IL-1β and IL-17 as well as on the increase in anti-inflammatory cytokine IL-10 in the murine colon [60]. Together, these data show that the biological activities of EPS are strain-dependent or depend on the structural diversity of EPS. Our results demonstrate, for the first time, that *Lg*-EPS are capable of increasing both AhR receptor expression and induction of AhR activity in HT29-Lucia™ AhR cells, known to be an immune regulator that can be activated by commensal bacteria or their microbial products. Among these products, it is well established that tryptophan metabolites and SCFAs are involved in regulating inflammation by mechanisms dependent on AhR activation, thereby contributing to intestinal homeostasis [58].

Investigation of the inhibitory properties of LAB of the intestinal microbiota on fungal pathogens is an interesting alternative to develop new therapies. As evidence of the mechanisms of *Lactobacillus-Candida* spp. interaction is scarce, it would be interesting to investigate the molecular determinants involved. The inhibitory capacity of *L. gasseri* strain LLG1 or its EPS on *C. albicans* was investigated in order to better understand the role of EPS in the interactions between this LAB and *Candida* spp. Confocal microscopy observations show close proximity between the two species. *L. gasseri* led to a significant decrease in biofilm formation by *Candida* after 24 h co-incubation. These results concur with those of a study examining the interactions between *C. albicans* and 20 *Lactobacillus* reference strains or clinical isolates. In this study, some strains such as *L. gasseri* had antagonistic activity against *C. albicans* in terms of virulence, notably *via* the inhibition of adhesion or biofilm formation [61]. Our data also correlate with a study on the inhibitory effects of *L. gasseri* on *Candida* spp. In that study, this LAB was capable of causing the death of yeasts, with viability that was reduced further in the presence of acetate [62]. Other *in vitro* studies have also reported the inhibitory activities of *Lactobacillus* spp. such as *L. gasseri* against *Candida* spp [63–65]. In the current study, *Lg*-EPS (2 mg) led to a significant decrease in *Candida* filamentation after 12 h, biofilm formation after 24 h and fungal adhesion on Caco-2 cells after 2 h. Furthermore, *Lg*-EPS slowed down fungal growth from 6 h of exposure (2 mg) or after 8 h exposure (1 mg). Few studies have analyzed the inhibitory properties of EPS from *Lactobacillus* on *Candida* spp. However, one study demonstrated that EPS purified from *L. rhamnosus GG* was capable of interfering with the growth, hyphae formation, and adhesion of *Candida* to epithelial cells [66]. Thus, probiotics of the genus *Lactobacillus* appear to exert their antimicrobial activity through the production of substances such as bacteriocins, hydrogen peroxide, organic acids, or fatty acids, as well as EPS [62–65,67]. A limitation of this study is that the concentrations of purified *Lg*-EPS required to observe biological effects *in* *vitro* may not directly reflect physiological concentrations encountered in gut environment. The concentration of purified Lactobacillus-derived EPS within the gastrointestinal tract remains largely unknown, making it difficult to directly relate in vitro exposure levels to physiological conditions. Previous studies investigating the immunomodulatory and antimicrobial properties of LAB-derived EPS have commonly employed concentrations ranging from several tens of µg/mL to 1 mg/mL, depending on the biological model and endpoint evaluated [68,69]. Therefore, the concentrations used in the present study should be regarded as mechanistic *in vitro* concentrations rather than direct estimates of gut exposure. Moreover, EPS produced by live bacteria may accumulate within localized microenvironments, further complicating direct comparisons between purified EPS concentrations and physiological exposure levels. Further studies will be required to quantify *Lg*-EPS levels *in vivo* and determine whether comparable biological effects occur under physiologically relevant conditions.

## Conclusion

EPS from *Lactobacilli* have many beneficial properties, including antioxidant, immunomodulatory, antitumour activity, or regulating intestinal homeostasis. *L. gasseri* LLG1 or *Lg*-EPS both have a protective and beneficial role through their anti-inflammatory properties, as well as a potential role as an adjuvant in antifungal strategies through their inhibitory activity on *C. albicans*. It would be interesting to carry out comparative analyses of the biological effects of EPS from several *L. gasseri* strains, which would allow for a better understanding of the effects specific to each strain compared to the conserved effects. It would also be interesting to study the structure of EPS in order to better understand the structure-activity relationship, particularly with regard to their beneficial properties.

## Supplementary Material

Supplementary Data.docx

## Data Availability

The data that support the findings of this study are openly available in “Figshare” at https://doi.org/10.6084/m9.figshare.30581381, reference number [35].
