# Supplementary material for: Activity of Lactobacillus gasseri strain LLG1 and its exopolysaccharides in an inflammatory cell model and on Candida albicans proliferation
Source: Virulence. 2026 Aug 28;17(1):2707757. doi: 10.1080/21505594.2026.2707757 (PMC13531990; doi:10.1080/21505594.2026.2707757)
Supplement: Supplementary file 1 [file KVIR_A_2707757_SM4886.docx]

**Supplementary DATA**

| **Human Gene** | **Primer Sequence (5’-3’)** | |
| --- | --- | --- |
| *GAPDH* | Forward | GAC-ACC-CAC-TCC-TCC-ACC-TTT |
|  | Reverse | TTG-CTG-TAG-CCA-AAT-TCG-TTG-T |
| *TLR2* | Forward | GCC-AAA-GCT-TTG-ATT-GAT-TGG |
|  | Reverse | TTG-AAG-TTC-AGC-TCC-TG |
| *TLR4* | Forward | CAG-GGC-TTT-TCT-GAG-TCG-TC |
|  | Reverse | TGA-GCA-GTC-GTG-CTG-GTA-TC |
| *NF-κB* | Forward | AGT-GAA-CCG-AAA-CTC-TGG-CA |
|  | Reverse | CCT-GGT-CCC-GTG-AAA-TAC-A |
| *Myd88* | Forward | GGA-ATG-TGA-CTT-CCA-GAC-CAA |
|  | Reverse | GAT-GGG-GAT-CAG-TCG-CTT-C |
| *AhR* | Forward | GCA-CCG-ATG-GGA-AAT-GAT-ACT-ATC |
|  | Reverse | GGT-GAC-CTC-CAG-CAA-ATG-AGT-T |
| *TNFα* | Forward | ATC-AAT-CGG-CCC-GAC-TAT-CTC |
|  | Reverse | ACA-GGG-CAA-TGA-TCC-CAA-AGT |
| *IL-6* | Forward | AGT-GAG-GAA-CAA-GCC-AGA-GC |
|  | Reverse | GTC-AGG-GGT-GGT-TAT-TGC-AT |
| *IL-8* | Forward | AAA-TCA-GGA-AGG-CTG-CCA-AGA |
|  | Reverse | AAG-GAA-CCA-TCT-CAC-TGT-GTG-TAA-AC |
| *IL-10* | Forward | ACT-TTA-AGG-GTT-ACC-TGG-GTT-GC |
|  | Reverse | TCA-CAT-GCG-CCT-TGA-TGT-CTG |
| *CCL2* | Forward | CCC-CAG-TCA-CCT-GCT-GTT-AT |
|  | Reverse | TGG-AAT-CCT-GAA-CCC-ACT-TC |
| *BAX* | Forward | CCT-TTT-GCT-TCA-GGG-TTT-CA |
|  | Reverse | AGA-CAC-TCG-CTC-AGC-TTC-TT |
| *Caspase 1* | Forward | AAA-AGC-CAT-GGC-CGA-CAA |
|  | Reverse | CTT-CCT-TGT-TCA-GCA-CCC-TT |
| *Caspase 9* | Forward | CGA-CCT-GAC-TGC-CAA-GAA-AA |
|  | Reverse | GAC-AGC-CGT-GAG-AGA-GAA-TG |

**Supplementary Table 1. Sequence of human primers used for q-PCR**

**ResultS**

**Supplementary Fig. 1. Evaluation of contaminants in *Lg*-EPS by SDS-PAGE, Western blot and spectrophotometry. (A)** Western blot analysis of lipoteichoic acid (LTA) in *Lg*-EPS or *L. gasseri*. Lane 1: 5 µL pre-stained protein marker; lane 2: 10 µg LTA (1 mg/mL) from *Staphylococcus* (positive control); lane 3: 20 µL of *L. gasseri* lysate (absorbance = 0.3 OD unit); lane 4: 20 µL *E. coli* lysate (absorbance = 0.3 OD unit; negative control); lane 5: 5 µg *Lg*-EPS (1 mg/mL); lane 6: 10 µg *Lg*-EPS (1 mg/mL); lane 7: 5 µg *Lg*-EPS + 10 µg LTA (both 1 mg/mL); lane 8: 10 µg *Lg*-EPS + 10 µg LTA (both 1 mg/mL). (**B**) SDS-PAGE analysis of protein in *Lg*-EPS. Lane 1: 5 µL pre-stained protein marker; lane 2: 20 µg BSA (1 mg/mL) (positive control); lane 3: 20 µg *Lg*-EPS (1 mg/mL). Data are representative of three independent experiments. (**C**) Spectrophotometric analysis of nucleic acids in *Lg*-EPS. (**1.**): spectrophotometric analysis of dsDNA; (**2.**): ssDNA; (**3.**): RNA in *Lg*-EPS. The purity of the nucleic acid samples was assessed using absorbance ratios: 260/280 nm and 260/230 nm. Conc.: concentration. Data represent three independent biological *Lg*-EPS samples, each analysed with one technical replicate.

**Supplementary Fig. 2. Cell proliferation assay by MTT and expression of various apoptotic markers expressed by Caco-2 cells in the presence of *Lg*-EPS.** Cell proliferation assay by MTT (%) at 24 h **(A)** and 48 h **(B)** using Caco-2 cells. Caco-2 represents the control group (Caco-2 cells alone); 250, 500, 1000 or 2000 correspond to Caco-2 treated with *Lg*-EPS at 250, 500, 1000 or 2000 µg/mL, respectively. **(C–E)** Relative expression levels of BAX, caspase-1 and caspase-9 mRNA in Caco-2 cells, respectively. Caco-2 represents the control group (Caco-2 cells alone); *Lg*-EPS corresponds to Caco-2 cells treated with EPS at 2000 µg/mL. Data represent three independent biological replicates per condition. Cell proliferation assay by MTT (**A-B**) were assessed in technical quintuplicates, whereas relative mRNA expression levels in Caco-2 cells (**C-E**) were assessed in technical quadruplicates.

**Supplementary Fig. 3. Confocal microscopy of *L. gasseri* and *C. albicans*, and co-incubation of *L. gasseri* and *C. albicans*.** (**A**) *L. gasseri* alone labelled with DAPI; (**B**) *C. albicans* alone labelled with ConA. Zoom of *L. gasseri* alone labelled with DAPI **(a)** or *C. albicans* alone labelled with ConA **(b)**. (**C1-C3**) Confocal microscopy of co-incubation of *L. gasseri* and *C. albicans*: *L. gasseri* labelled with DAPI and co-incubated with *C. albicans* (**C1**); *C. albicans* labelled with ConA and co-incubated with *L. gasseri* (**C2**); (**C3**) merged images of (**C1**) and (**C2**). (**c1-c2**) Zoom of *L. gasseri* labelled with DAPI and co-incubated with *C. albicans* labelled with ConA. The scale bars correspond to 10 μm or 5 µm for zoom images. Data are representative of three independent experiments.
